# NovoTTF^™^-100A System (Tumor Treating Fields) transducer array layout planning for glioblastoma: a NovoTAL^™^ system user study

**DOI:** 10.1186/s12957-015-0722-3

**Published:** 2015-11-11

**Authors:** Aafia Chaudhry, Laura Benson, Michael Varshaver, Ori Farber, Uri Weinberg, Eilon Kirson, Yoram Palti

**Affiliations:** Medical Affairs, Novocure Ltd., New York, NY USA; Technical Operations, Novocure Ltd., 195 Commerce Way, Portsmouth, NH USA; Research and Development, Novocure Ltd., Topaz Building, 4th Floor, MATAM Center, PO Box 15022, Sha’ar HaCarmel, Haifa Israel; Clinical Development, Novocure Ltd., Park 6, CH-6039 Root D4, Lucerne, Switzerland

**Keywords:** Glioblastoma, Optune, NovoTAL System, NovoTTF-100A System, Tumor Treating Fields, TTFields

## Abstract

**Background:**

Optune™, previously known as the NovoTTF-100A System™, generates Tumor Treating Fields (TTFields), an effective anti-mitotic therapy for glioblastoma. The system delivers intermediate frequency, alternating electric fields to the supratentorial brain. Patient therapy is personalized by configuring transducer array layout placement on the scalp to the tumor site using MRI measurements and the NovoTAL System. Transducer array layout mapping optimizes therapy by maximizing electric field intensity to the tumor site. This study evaluated physician performance in conducting transducer array layout mapping using the NovoTAL System compared with mapping performed by the Novocure in-house clinical team.

**Methods:**

Fourteen physicians (7 neuro-oncologists, 4 medical oncologists, and 3 neurosurgeons) evaluated five blinded cases of recurrent glioblastoma and performed head size and tumor location measurements using a standard Digital Imaging and Communications in Medicine reader. Concordance with Novocure measurement and intra- and inter-rater reliability were assessed using relevant correlation coefficients. The study criterion for success was a concordance correlation coefficient (CCC) >0.80.

**Results:**

CCC for each physician versus Novocure on 20 MRI measurements was 0.96 (standard deviation, SD ± 0.03, range 0.90–1.00), indicating very high agreement between the two groups. Intra- and inter-rater reliability correlation coefficients were similarly high: 0.83 (SD ±0.15, range 0.54–1.00) and 0.80 (SD ±0.18, range 0.48–1.00), respectively.

**Conclusions:**

This user study demonstrated an excellent level of concordance between prescribing physicians and Novocure in-house clinical teams in performing transducer array layout planning. Intra-rater reliability was very high, indicating reproducible performance. Physicians prescribing TTFields, when trained on the NovoTAL System, can independently perform transducer array layout mapping required for the initiation and maintenance of patients on TTFields therapy.

## Background

**Fig. 1:**
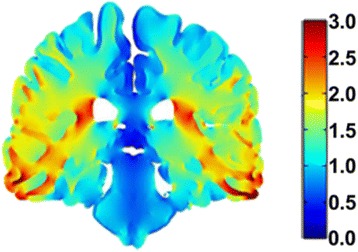
Electric field magnitude and distribution (in V/cm) shown in coronal view from a finite element method simulation model. This simulation employs a left-right paired transducer array configuration. Reprinted with permission from Miranda et al. [5]

**Fig. 2:**
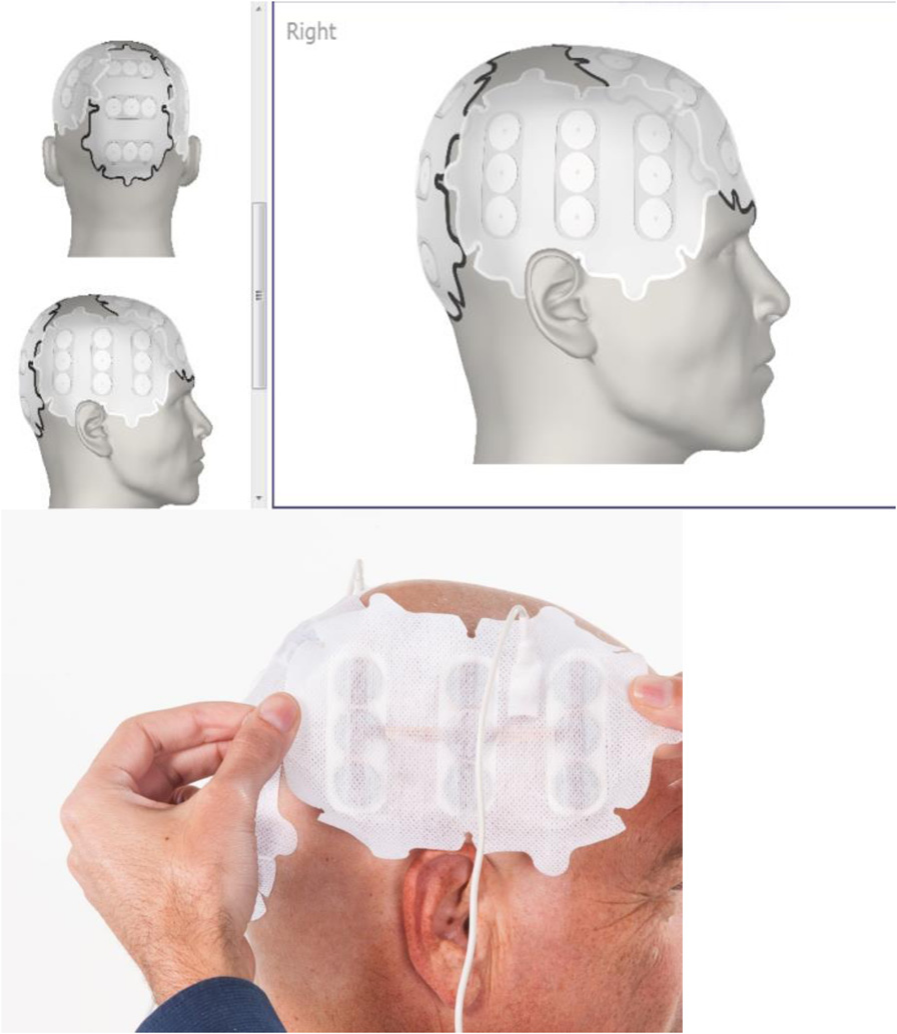
Sample transducer array layout map guiding placement of transducer arrays on the scalp

NovoTTF-100A System (Optune^™^, Novocure Ltd., Haifa, Israel) is a unique modality of anti-cancer therapy delivering specifically tuned alternating electric fields, Tumor Treating Fields (TTFields), and is approved by the US Food and Drug Administration (FDA) for the treatment of patients with glioblastoma multiforme (GBM). Initial approval was based on the results of a large, randomized, phase 3 study demonstrating comparable efficacy and favorable safety as monotherapy, compared with the best physician’s choice of active chemotherapy in patients with recurrent GBM [1]. TTFields evolved from preclinical observations that alternating electric fields could selectively arrest cellular division in cancer cells by impairing normal mitosis and cytokinesis. The effects of TTFields, which have been described previously, include interference with normal mitotic spindle assembly during metaphase, cellular membrane disruption, cytoplasmic blebbing during anaphase, aberrant chromosomal segregation, and intracellular dielectrophoresis of polar macromolecules, leading to the destruction of the cleavage furrow, during cytokinesis [2–4]. As the effects of TTFields are selective for dividing cells, quiescent cells in the normal human brain are spared. TTFields possess directional specificity, frequency dependence, and intensity dependence in exerting a therapeutic effect across different cell lines. In glioma cells, maximal inhibition of cellular proliferation was observed at a frequency of 200 kilohertz (kHz), and this effect increased with higher electric field intensities [2]. Complete arrest of cellular proliferation in cultures occurred when electric field intensity reached 2.25 volts per centimeter (V/cm). In addition, the effect of TTFields is directly proportional to the orientation of the mitotic axis relative to electric field direction. Cellular damage is maximal when the axis of division is aligned with the direction of the electric field and is minimal when the cleavage axis is orthogonal to the field direction [2]. By applying electric fields in multiple directions, an additive cytotoxic effect can be obtained [3]. To characterize how electric fields behave and distribute within the human head, modeling frameworks based on anatomical head models using Finite Element Method (FEM) simulations have been developed [5]. These simulations yield realistic head models based on magnetic resonance imaging (MRI) measurements and compartmentalize tissue types such as skull, white matter, gray matter, and cerebrospinal fluid (CSF) within the head. Each tissue type is assigned dielectric properties for relative conductivity and permittivity, and simulations are run whereby different transducer array configurations are applied to the surface of the model in order to understand how an externally applied electric field, of preset frequency, will distribute throughout the brain. The results of these simulations, employing paired array configurations, a constant current, and a preset frequency of 200 kHz, have demonstrated that electric field distributions are relatively nonuniform throughout the brain and that electric field intensities exceeding 1 V/cm are generated in most tissue compartments except CSF [5]. These results are obtained assuming total currents with a peak-to-peak value of 1800 milliamperes (mA) at the transducer array-scalp interface. This threshold of electric field intensity is sufficient to arrest cellular proliferation in glioblastoma cell lines. Additionally, by manipulating the configuration of paired arrays, it is possible to achieve an almost tripling of electric field intensity to a particular region of the brain (Fig. 1). The NovoTAL^™^ System (Novocure Ltd., Haifa, Israel) is used to guide the optimal transducer array layout for a patient based on the location and extent of their contrast-enhancing tumor. Initial morphometric head size measurements are determined from the T1 sequences of a brain MRI, using axial and coronal views. Postcontrast axial and coronal MRI slices are selected to demonstrate the maximal diameter of enhancing lesions. Employing measures of head size and distances from predetermined fiducial markers to tumor margins, the system runs permutations and combinations of paired array layouts in order to generate the configuration which delivers maximal electric field intensity to the tumor site. The output is a three-dimensional array layout map, which is used by the patient and caregiver in arranging arrays on the scalp during the normal course of TTFields therapy (Fig. 2). This user study aimed to assess TTFields certified prescribing physician competency in autonomously performing the standard measurements required for initiating and maintaining patients on optimal therapy.

## Methods

In January 2013, 14 physicians (7 neuro-oncologists, 4 medical oncologists, and 3 neurosurgeons) were randomly selected from the pool of NovoTTF-100A System certified prescribing sites. All physicians provided written informed consent to participate in the study. The participants represented approximately 20 % of certified sites at the time of this study. The physicians were representative of the TTFields therapy certified prescriber base and covered the core specialties that may be involved in standard GBM treatment planning.

Each physician had to complete mapping measurements on a total of five distinct MRI cases. The MRI measurements were obtained using the same standard Digital Imaging and Communications in Medicine (DICOM) viewer (MicroDicom). The MRIs were anonymized to protect patient privacy and included scans with the following anatomical lesions: a right fronto-temporal tumor, a right parieto-temporal tumor, a left fronto-temporal tumor, a left parieto-occipital tumor, and a multi-focal midline tumor. Test case MRIs were randomly selected from a pool of scans featuring tumors in the same anatomical locations. A standardized NovoTAL measurement case report form was provided outlining the required measurements to be obtained for each case. All measurements commenced from fiducial markers at the outer margin of the scalp and extended tangentially from a right-, anterior-, superior origin.

**Fig. 3:**
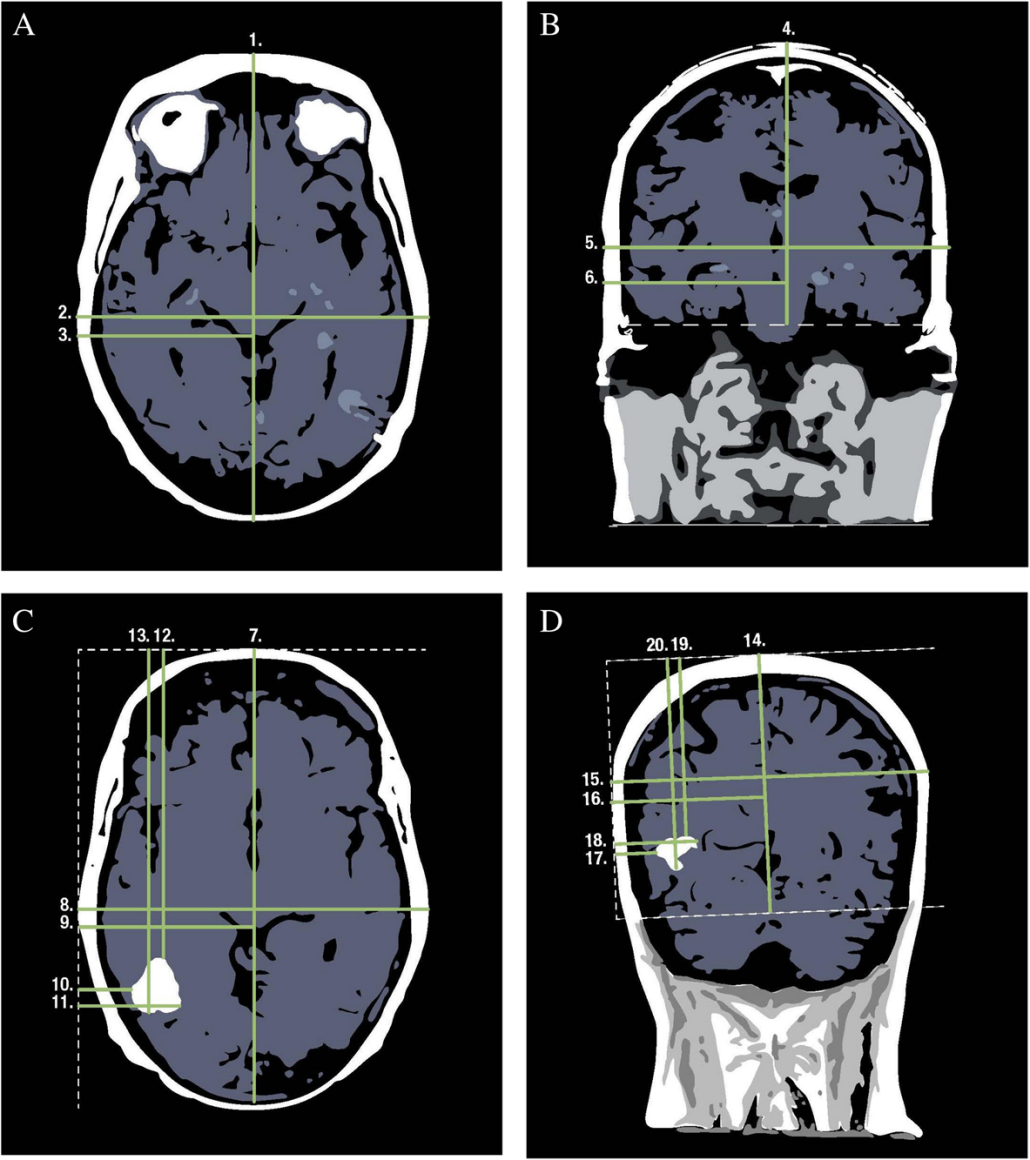
Magnetic resonance imaging. **a** Axial T1 sequence slice containing most apical image, including orbits used to measure head size. **b** Coronal T1 sequence slice selecting image at level of ear canal used to measure head size. **c** Postcontrast T1 axial image shows maximal enhancing tumor diameter used to measure tumor location. **d** Postcontrast T1 coronal image shows maximal enhancing tumor diameter used to measure tumor location

Firstly, morphometric head size was estimated from the axial T1 MRI sequence selecting the most apical image which still included the orbits (or the image directly above the superior edge of the orbits) (Fig. 3a).

A total of 20 measurements were required to map each distinct case:

1. Maximal antero-posterior (A-P) head size, commencing measurement from the outer margin of the scalp.
2. Maximal width of the head perpendicular to the A-P measurement: right to left lateral distance.
3. Distance from the far most right margin of the scalp to the anatomical midline.

Coronal view head size measurements were obtained on the T1 MRI sequence selecting the image at the level of the ear canal (Fig. 3b).

The following coronal head size measurements were obtained:

4. A vertical measurement from the apex of the scalp to an orthogonal line delineating the inferior margin of the temporal lobes.
5. Maximal right to left lateral head width.
6. Distance from the far right margin of the scalp to the anatomical midline.

Measurements for tumor location were made in the following manner using T1 postcontrast MRI sequences, firstly on the axial image demonstrating maximal enhancing tumor diameter (Fig. 3c):

7. The maximal A-P head size, excluding the nose.
8. The maximal right to left lateral diameter, measured perpendicular to the A-P distance.
9. Distance from the right margin of the scalp to the anatomical midline.
10. Distance from the right margin of the scalp to the closest tumor margin, measured parallel to the right-left lateral distance and perpendicular to the A-P measurement.
11. Distance from the right margin of the scalp to the farthest tumor margin, measured parallel to the right-left lateral distance, perpendicular to the A-P measurement.
12. Distance from the front of the head, measured parallel to the A-P measurement, to the closest tumor margin.
13. Distance from the front of the head, measured parallel to the A-P measurement, to the farthest tumor margin.

Coronal view tumor measurements were completed identifying the postcontrast T1 MRI slice featuring the maximal diameter of tumor enhancement (Fig. 3d).

14. The maximal distance from the apex of the scalp to the inferior margin of the cerebrum. In anterior slices, this would be demarcated by a horizontal line drawn at the inferior margin of the frontal or temporal lobes, and posteriorly, it would extend to the lowest level of visible tentorium.
15. Maximal right to left lateral head width.
16. Distance from the right margin of the scalp to the anatomical midline.
17. Distance from the right margin of the scalp to the closest tumor margin, measured parallel to the right-left lateral distance.
18. Distance from the right margin of the scalp to the farthest tumor margin, measured parallel to the right-left lateral distance.
19. Distance from the apex of the head to the closest tumor margin, measured parallel to the superior apex to inferior cerebrum line.
20. Distance from the apex of the head to the farthest tumor margin, measured parallel to the superior apex to inferior cerebrum line.

All measurements were rounded to the nearest millimeter and were entered into the NovoTAL case report form and software for analysis. Gold standard measurements performed by Novocure in-house clinical staff were collected for all five cases and were used as the comparator benchmark.

### Statistical analysis

Degree of agreement between user study participants and Novocure gold standard measurements were assessed for each individual physician and for each individual case using the concordance correlation coefficient (CCC) method using the Statistical Analysis System (SAS) version 9.3 software. Intragroup and intergroup agreement was assessed using the Shourt and Fliess’ intra-rater (ICC(3,1)) and inter-rater (ICC(2,1)) concordance correlation methods [6]. Overall, for the user study to be positive on the primary analysis, the average physician-company agreement required a CCC >0.80, performed with an accuracy threshold of ±7.5 mm. This threshold represents approximately 0.001 % of the wavelength of 200 kHz TTFields. For the secondary analyses, intragroup agreement, ICC(3,1), and intergroup agreement, ICC(2,1), values >0.80 would indicate near perfect concordance, and values in the range of 0.61–0.80 would indicate substantial agreement.

## Results

**Fig. 4:**
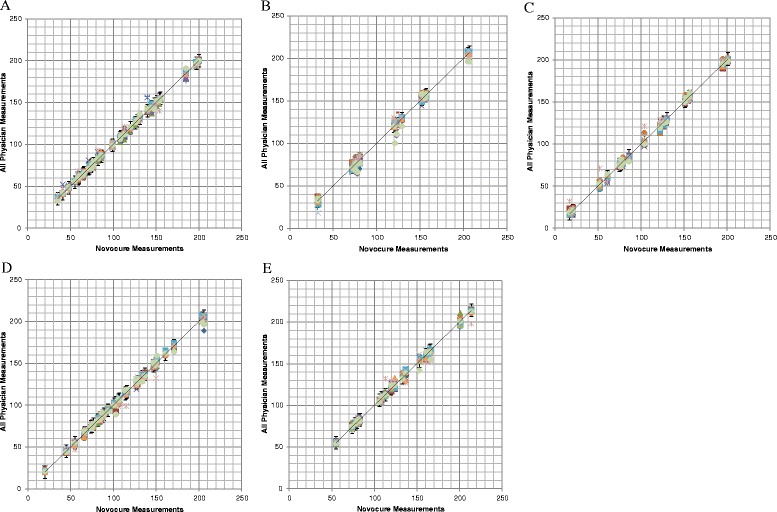
Scatterplots and linear correlation between Novocure and physician magnetic resonance imaging (MRI) measurement for MRI cases 1 to 5. *Error bars* represent ±7.5 mm from Novocure measurements. *Each color* represents a set of measurements made by a different physician (*n* = 14). Mean (SD) *R* ^2^ linear correlations for all physicians versus Novocure were **a** case 1, 0.9966 (0.0039); **b** case 2, 0.9954 (0.0041); **c** case 3, 0.9967 (0.0050); **d** case 4, 0.9963 (0.0037); and **e** case 5, 0.9935 (0.0066)

Data were collected from all 14 user study participants in January 2013. All physicians obtained the 20 required measurements for each of the five distinct MRI cases, and there were no missing data. Pairwise concordance between the study participants and Novocure gold standard measurements was assessed for each physician case (Fig. 4). CCC was calculated for each physician versus gold standard measures on 20 unique data points and then averaged. The average CCC was 0.88 (SD 0.11). Using the predetermined threshold of ±7.5 mm for each of the 20 measurements, the average physician agreement with gold standard was 0.96 (SD 0.03, range 0.90–1.00). Therefore, the study was positive for the primary analysis (Table 1). The intra-rater reliability for the 14 physicians across the five individual MRI cases was assessed using the intraclass correlation coefficient ICC(2,1). The 20 measurement points from each MRI case were averaged to perform the calculation, demonstrating an average ICC(3,1) of 0.80 (SD 0.18, range 0.48–1.00), thus showing excellent intra-rater reliability in performing the key measurements required for TTFields therapy planning. The inter-rater reliability ICC(2,1) was similarly high, at 0.80.

**Table 1:** Summary of average concordance correlation coefficient between Novocure and physicians for MRI test cases by tumor position

Test case	Tumor position	Average concordance correlation coefficient (CCC) *R* ^2^ ± SD
1	Right fronto-temporal	0.9966 ± 0.0039
2	Right parieto-occipital	0.9954 ± 0.0041
3	Left fronto-temporal	0.9967± 0.0050
4	Left parieto-occipital	0.9963 ± 0.0037
5	Midline, multi-focal	0.9935 ± 0.0066

## Discussion

The results of this user study have demonstrated for the first time that irrespective of clinical specialty, physicians prescribing TTFields for patients with GBM can independently perform the measurements required for treatment initiation and maintenance with the same degree of accuracy as gold standard mapping and planning currently performed by Novocure clinicians. The concordance between in-house clinicians and study participants was very high, as was intra-rater reliability. The positive results of this user study led to the submission of a supplement to the premarket approval application (PMA) being approved by the FDA in August 2013. The PMA supplement approval provided authorization for transferring the responsibility for performing NovoTAL transducer array mapping from Novocure to trained physicians prescribing TTFields. Until recently, all personalized transducer array layout maps, which are prerequisite for treatment initiation with TTFields, were generated by Novocure in-house clinical staff. However, there are many clinical advantages in transferring the process of array layout mapping to the prescribing physician. The prescribing physician can better correlate radiographic findings with a patient’s clinical status and at their own discretion, better evaluate which specific areas of enhancing lesion to focus the TTFields toward. Since the recent inclusion of TTFields to the clinical armamentarium is for patients with newly diagnosed aswell as recurrent GBM, reports are emerging in the literature that describe “out of field” tumor recurrences that subsequently respond to therapy following adjustment of the transducer array configuration, based on evidence of radiographic recurrence [7]. In one case, a 41-year-old man who underwent gross total resection for a left frontal GBM with primitive neuroectodermal (PNET) component subsequently received standard adjuvant radiochemotherapy followed by a combination of TTFields with metronomic temozolomide. At 6 months, a new distal lesion was noted in the left parietal lobe. Following a further resection, readjustment of the transducer array configuration to target the distal parietal lesion, and a change to his chemotherapy regimen, the patient was able to continue on TTFields therapy for more than a year. Cases such as these suggest that local tumor progression may respond to adjustment of TTFields when there are imaging changes.

Interpreting radiographic changes in response to GBM therapy can be complicated by the use of agents that impact the blood brain barrier, impeding uptake of contrast, such as anti-angiogenic therapy [8–10]. As prescribing clinicians have comprehensive information on a patient’s clinical history and prior therapies received in the course of their GBM management, they are better placed to interpret which MRI features are likely to represent true active tumor, a clinical response, pseudo-response, stable disease, or frank progression over a series of scans. This may lead to a more informed approach when planning TTFields treatment and may ultimately help improve patient outcomes.

## Conclusions

This user study demonstrated a very high degree of concordance between prescribing physicians and the in-house Novocure clinical team in performing transducer array layout planning for patients with recurrent GBM. Intra-rater reliability was very high, indicating that physician performance was reproducible. Physicians prescribing the TTFields, when trained on the NovoTAL System, should be able to independently perform transducer array layout mapping required for the initiation and maintenance of patients receiving TTFields. Adjusting the configuration of transducer arrays in response to changes in a patient’s imaging may impact clinical outcomes. Prospective studies are warranted to determine the optimal timing at which a new transducer array layout map should be generated for patients receiving ongoing therapy with TTFields.

## Abbreviations

A-P: antero-posterior
CCC: concordance correlation coefficient
CSF: cerebrospinal fluid
DICOM: Digital Imaging and Communications in Medicine
FEM: Finite Element Method
GBM: glioblastoma multiforme
ICC(2,1): Shourt and Fliess’ inter-rater concordance correlation method
ICC(3,1): Shourt and Fliess’ intra-rater concordance correlation method
kHz: kilohertz
mA: milliamperes
MRI: magnetic resonance imaging
PMA: premarket approval application
PNET: primitive neuroectodermal tumor
SAS: Statistical Analysis System
SD: standard deviation
TTFields: Tumor Treating Fields
US FDA: United States food and drug administration
V/cm: volts per centimeter
